# A Five-microRNA Signature for Survival Prognosis in Pancreatic Adenocarcinoma based on TCGA Data

**DOI:** 10.1038/s41598-018-22493-5

**Published:** 2018-05-16

**Authors:** Xiu-Hui Shi, Xu Li, Hang Zhang, Rui-Zhi He, Yan Zhao, Min Zhou, Shu-Tao Pan, Chun-Le Zhao, Ye-Chen Feng, Min Wang, Xing-Jun Guo, Ren-Yi Qin

**Affiliations:** Department of Biliary-Pancreatic Surgery, Affiliated Tongji Hospital, Tongji Medical College, Huazhong University of Science and Technology, Wuhan, China

## Abstract

Novel biomarkers for pancreatic adenocarcinoma are urgently needed because of its poor prognosis. Here, by using The Cancer Genome Atlas (TCGA) RNA-seq data, we evaluated the prognostic values of the differentially expressed miRNAs and constructed a five-miRNA signature that could effectively predict patient overall survival (OS). The Kaplan-Meier overall survival curves of two groups based on the five miRNAs were notably different, showing overall survival in 10.2% and 47.8% at five years for patients in high-risk and low-risk groups, respectively. The ROC curve analysis achieved AUC of 0.775, showing good sensitivity and specificity of the five-miRNA signature model in predicting pancreatic adenocarcinoma patient survival risk. The functional enrichment analysis suggested that the target genes of the miRNA signature may be involved in various pathways related to cancer, including PI3K-Akt, TGF-β, and pluripotent stem cell signaling pathways. Finally, we analyzed expression of the five specific miRNAs in the miRNA signature, and validated the reliability of the results in 20 newly diagnosed pancreatic adenocarcinoma patients using qRT-PCR. The expression results of qRT-PCR were consistent with the TCGA results. Taken together, these findings suggested that the five-miRNA signature (hsa-miR-203, hsa-miR-424, hsa-miR-1266 hsa-miR-1293, and hsa-miR-4772) could be used as a prognostic marker for pancreatic adenocarcinoma.

## Introduction

Pancreatic adenocarcinoma is the most common type of cancers in the pancreas and is the fourth leading cause of cancers deaths^1^. The expected 5 year survival rate is less than 5%, a statistic that has remained largely unchanged for the past forty years^2^. Pancreatic adenocarcinoma patients are characterized by a lack of clinical manifestations until the late stages of the disease, leading to a poor prognosis and high mortality rate. At the time of diagnosis, only approximately 8% of pancreatic adenocarcinomas are localized and considered resectable^3^. Many of the proteins used as biomarkers for pancreatic adenocarcinoma are also expressed in patients with chronic pancreatitis, leading to a decrease in the marker’s specificity for the diagnosis of pancreatic adenocarcinoma^4^. Complicated by these factors, the currently available pancreatic adenocarcinoma biomarkers have relatively low sensitivities and specificities that limit the scope of their clinical applications. Therefore, there is an urgent need to develop novel biomarkers or models for survival risk prediction in pancreatic adenocarcinoma that would provide patients with more effective therapies.

MicroRNAs (miRNAs) are small non-coding RNAs of 18–25 nucleotides in length that regulate gene expression by binding to the 3′-untranslated region(3′-UTR) of their target mRNAs, resulting in mRNA degradation and/or inhibition of mRNA translation^5^. miRNAs regulate the expression of more than 30% of human genes, which implies they play key roles in many biological function^6^. It was reported that a number of miRNAs have been identified as predictors of the clinical outcome in pancreatic adenocarcinoma^7^. However, due to the small patient number, limited number of miRNAs investigated, or different miRNA-chip platform, the studies lacked a normalized standard. Therefore, a larger patient cohort and normalization controls, as well as a standard protocol for more specific prognostic classifiers, are warranted.

TCGA is a National Cancer Institute effort to profile at least twenty different tumor types using genomic platforms and to make raw and processed data available to all researchers^8^. The TCGA has released a large amount of miRNA sequencing data from pancreatic adenocarcinoma patients. The aim of this study was to identify the differential miRNA expression patterns between pancreatic adenocarcinoma tissues and matched normal pancreatic tissues by analyzing the high-throughput miRNA data downloaded from TCGA database. Additionally, we evaluated the prognostic value of the differentially expressed miRNAs and constructed a five-miRNA signature that could effectively predict patient survival.

**Table 1:** Information on 10 candidate miRNAs associated with OS of pancreatic adenocarcinoma patients.

Gene symbol	HR	z-score	P-value
hsa-mir-1293	1.382070384	3.936761186	8.26E-05
hsa-mir-424	1.806200657	3.932111677	8.42E-05
hsa-mir-203	1.235918659	3.628114208	0.0002855
hsa-mir-181b-2	1.410660924	2.991217943	0.00277867
hsa-mir-1266	1.356523732	2.876138745	0.00402573
hsa-mir-100	1.252643694	2.79022654	0.00526712
hsa-mir-196b	1.137286841	2.778414113	0.0054625
hsa-mir-196a-1	1.133492951	2.673269706	0.00751158
hsa-mir-196a-2	1.128319707	2.640584119	0.00827632
hsa-mir-4772	1.240022579	2.58277929	0.0098008

*Derived from the univariate Cox regression analysis. HR: hazard ratio.

## Results

### Establishment of a five-miRNA signature associated with overall survival of pancreatic adenocarcinoma patients

**Figure 1:**
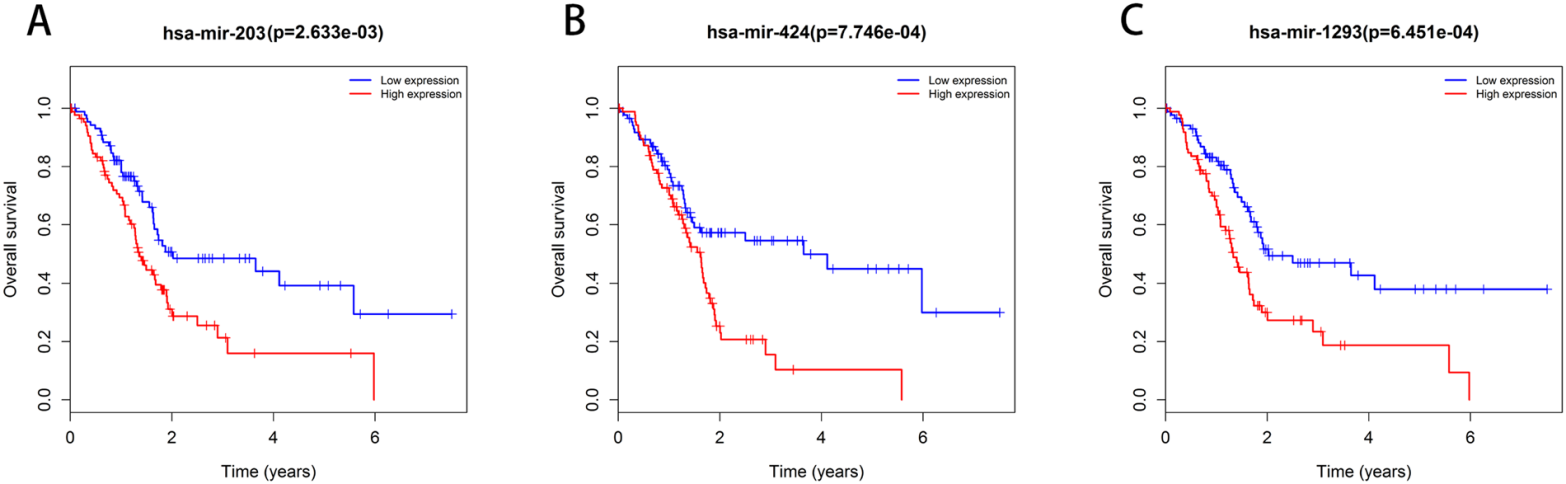
Three miRNAs that act as independent prognostic biomarkers were associated with overall survival in pancreatic adenocarcinoma patients. The patients were stratified into a high-risk group and a low-risk group according to median of each miRNA. (**A**) has-mir-203. (**B**) has-mir-424. (**C**) has-mir-1293.

To identify prognosis-related miRNAs, we first used the univariate Cox regression analysis to evaluate the associations between the expression level of each of the 50 differentially expressed miRNAs (DEMis) that were screened in our previous study and patients’ overall survival, and found that 10 miRNAs (Table 1) were significantly related to overall survival (p < 0.01). Then, a stepwise multivariate Cox regression analysis was performed, and five of the initial 10 miRNAs (Table 2) were selected to establish a predictive model. As previously described, the predictive model was defined as the linear combination of the expression levels of the five miRNAs weighted by their relative coefficients in the multivariate Cox regression test as follows: survival risk score(SRS) = (0.2250 × expression value of hsa-mir-203) + (0.5653 × expression value of hsa-mir-424) + (0.1771 × expression value of hsa-mir-1266) + (0.1937 × expression value of hsa-mir-1293) + (0.1846 × expression value of hsa-mir-4772). The results of the multivariate Cox regression analysis suggested that three miRNAs, hsa-mir-203(Fig. 1A), hsa-mir-424(Fig. 1B), and hsa-mir-1293(Fig. 1C) can act as independent prognostic factor in pancreatic adenocarcinoma (p < 0.05). All five miRNAs showed positive coefficients in the Cox regression analysis, indicating high-risk signatures for these five miRNAs since their high expression levels indicated a shorter overall patient survival.

**Table 2:** Information on the 5 prognostic miRNAs associated with OS of pancreatic adenocarcinoma patients.

Gene symbol	RC	Z-score	P-value
hsa-mir-203	0.225	2.74	0.0062
hsa-mir-424	0.5653	3.18	0.0015
hsa-mir-1266	0.1771	1.52	0.1293
hsa-mir-1293	0.1937	2.15	0.0314
hsa-mir-4772	0.1846	1.88	0.06

*Derived from the multivariate Cox regression analysis. RC: Relative coefficient.

### Risk stratification and ROC curve indicate good performance of the five-miRNA signature in predicting the overall survival of pancreatic adenocarcinoma patients

**Figure 2:**
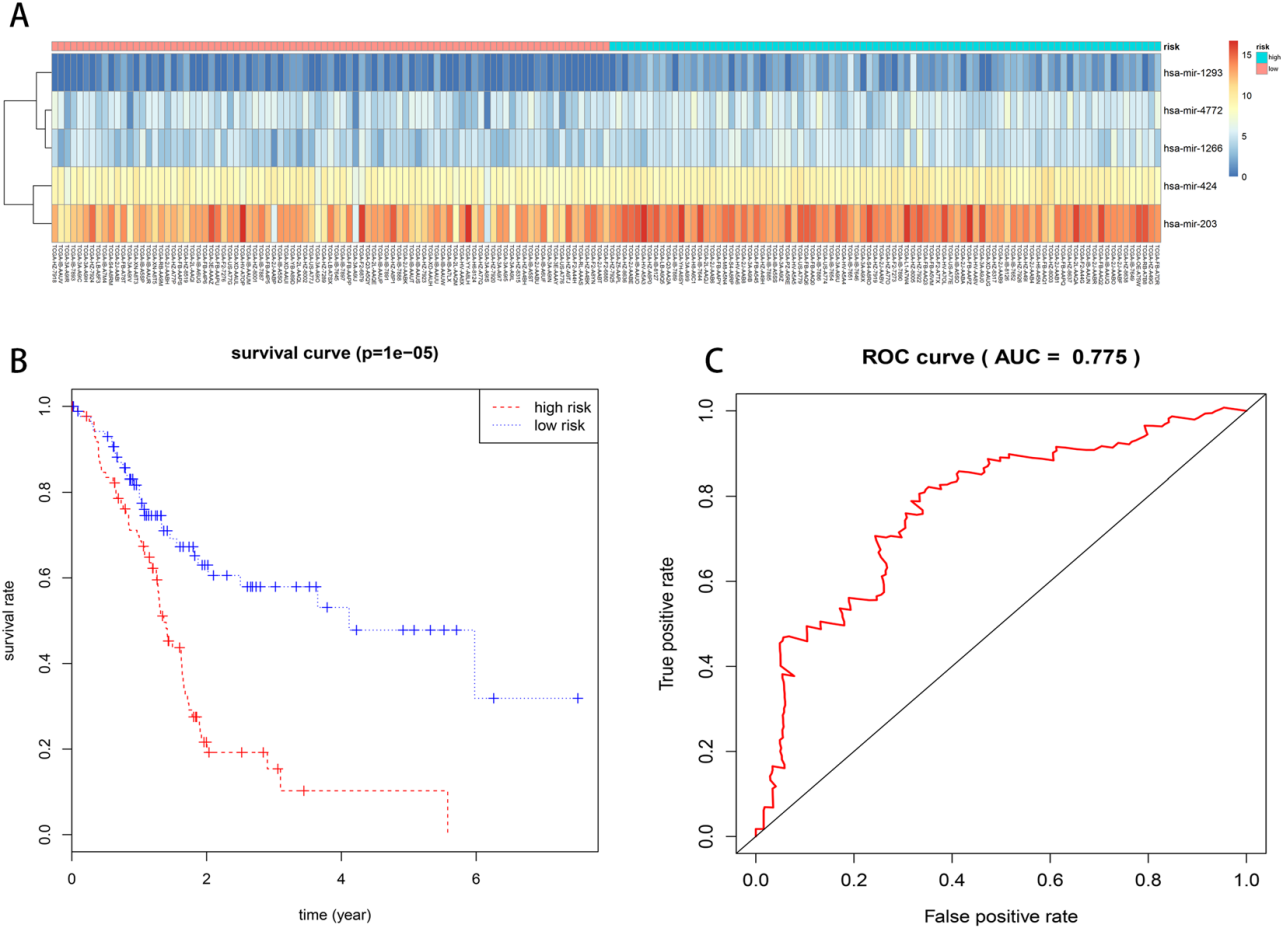
Prognostic evaluation of the five-miRNA signature in pancreatic adenocarcinoma patients. (**A**) The expression heatmap of 5 prognostic miRNAs. (**B**) Kaplan-Meier survival curve analysis for overall survival of pancreatic adenocarcinoma patients using the five-miRNA signature. (**C**) ROC curve analysis of the five-miRNA signature.

For each of the 177 patients in our study, we were able to calculate a five-miRNA expression-based survival risk score and assigned them into a high-risk group (n = 88) or a low-risk group (n = 89) according to the median risk score (Fig. 2A). The Kaplan-Meier overall survival curves of the two groups based on the five miRNAs were notably different (log-rank p = 1^e-05^ < 0.001), showing overall survival in 10.2% (95% CI, 0.0357–0.294) and 47.8% (95% CI, 0.338–0.677) at five years for patients with high-risk and low-risk SRS, respectively (Fig. 2B). The prognostic power of the five-miRNA signature was evaluated by calculating the AUC of ROC curve. A higher AUC indicates better model performance and an AUC of more than 0.70 is considered good performance. In our study, the ROC curve analysis achieved an AUC value of 0.775, showing good sensitivity and specificity of the five-miRNA signature model in predicting pancreatic adenocarcinoma patient survival risk (Fig. 2C).

### The target gene prediction of the prognostic five-miRNA signature

**Figure 3:**
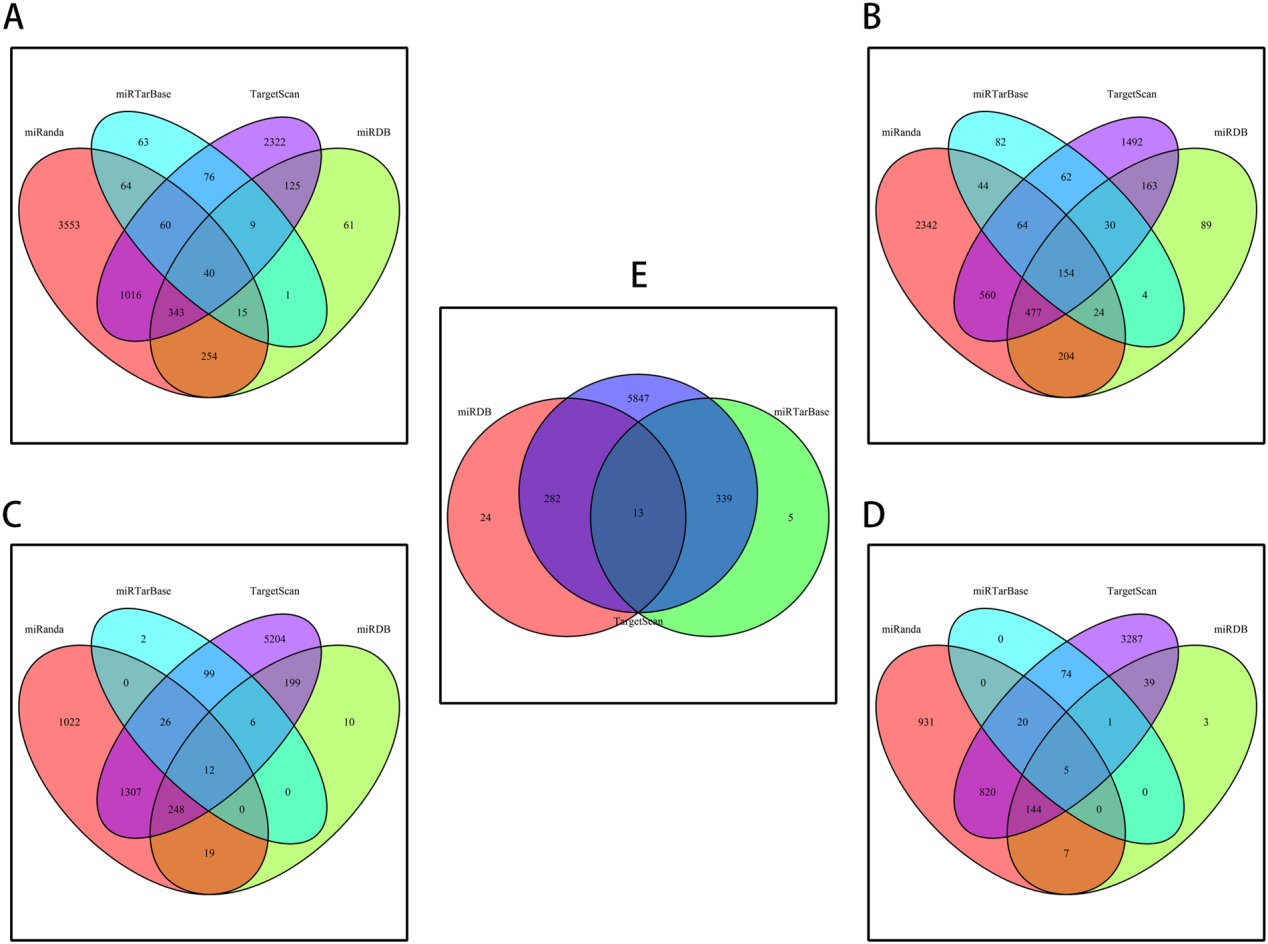
Prediction of the genes targeted the five-miRNA signature. The overlapping target genes were predicted using the miRanda, miRTarBase, TargetScan, and miRDB online analysis tools. (**A**) miRNA-203. (**B**) miRNA-424. (**C**) miR-1266. (**D**) miRNA-1293. (**E**) The overlapping target genes of miRNA-4772 were predicted using the miRTarBase, TargetScan, and miRDB online analysis tools.

The target genes of prognostic miRNAs were predicted using TargetScan^9^, miRDB^10^, miRanda^11^, and miRTarBase^12^ analysis tools. To further enhance the reliability of the bioinformatic analysis, the overlapping target genes were identified using Venn diagram^13^ (Fig. 3). The results indicated that 40, 154, 12, and 5 overlapping genes were identified for miR-203, miR-424, miR-1266, and miR-1293, respectively by the four databases above. Thirteen overlapping genes were identified for miR-4772 by TargetScan, miRDB, and miRTarBase. Compared with the other four, miR-4772 is a more recently identified miRNA and its targets could not be predicted using the miRanda database. In total, a list of 224 target genes was generated for the prognostic miRNA signature, but 5 of them overlap.

### GO annotation and KEGG signaling pathway analyzed predicted target genes of the prognostic five-miRNA signature

**Figure 4:**
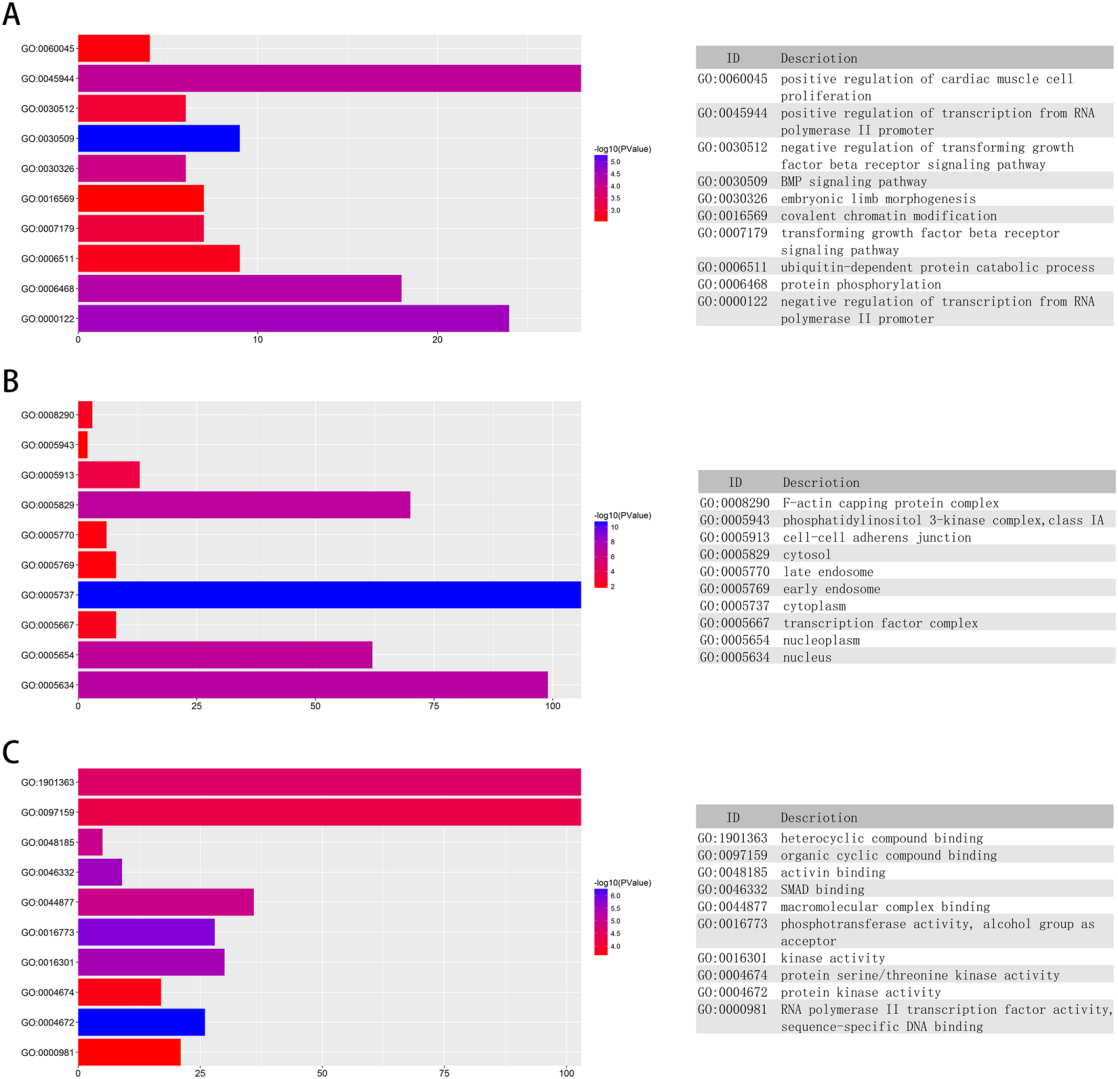
GO annotation analysis of the target genes. (**A**) Biological process (BP). (**B**) Cellular Component (CC). (**C**) Molecular Function (MF).

**Figure 5:**
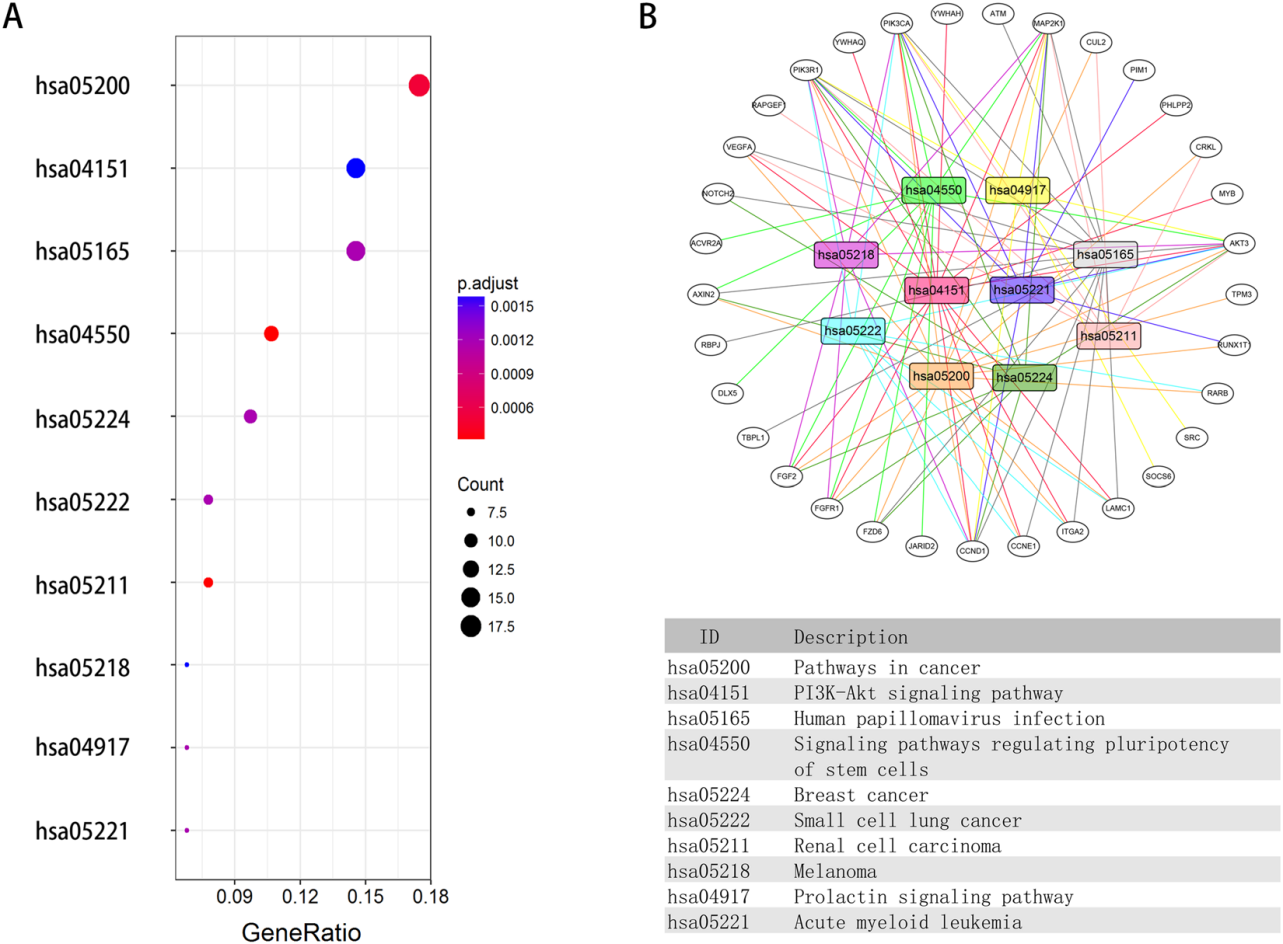
KEGG pathway functional enrichment analysis of the target genes. (**A**) The top ten pathways from the KEGG pathway functional enrichment analysis. (**B**) Gene-concept networks from the KEGG analysis; the colored rectangles represent pathways and the white ovals represent genes.

To further explore the potential biological function and mechanism of the five-miRNA signature, we analyzed the 219 potential targets described above through GO annotation and KEGG signaling pathway analysis. The GO annotation and KEGG signaling pathway were analyzed by two bioinformatics tools. The Database for Annotation, Visualization and Integrated Discovery (DAVID)^14^ and KOBAS^15^, respectively. The results of GO annotation and KEGG pathway analysis are listed in supplemental Tables 1 and 2, respectively. A p-value < 0.05 was set as the cut-off criteria. We have showed top ten terms from the GO results: biological process (Fig. 4A), cellular component (Fig. 4B), and molecular function (Fig. 4C). In these three categories, we found that many functions were primarily associated with molecular binding and gene transcription. Therefore, the five miRNAs might be involved with gene expression and the cellular and biological functions. The top ten pathways from the KEGG results are displayed in Fig. 5A. The results indicate that cancer-related pathways are obviously activated, including melanoma, breast cancer, small cell lung cancer, and renal carcinoma. In addition, to provide a readable graphic representation of the complex relationship between target genes and relative KEGG pathway, the “pathway-gene network” was constructed by Cytoscape^16^ (Fig. 5B).

### Functional implication of the prognostic five-miRNA signature PPI network construction

**Figure 6:**
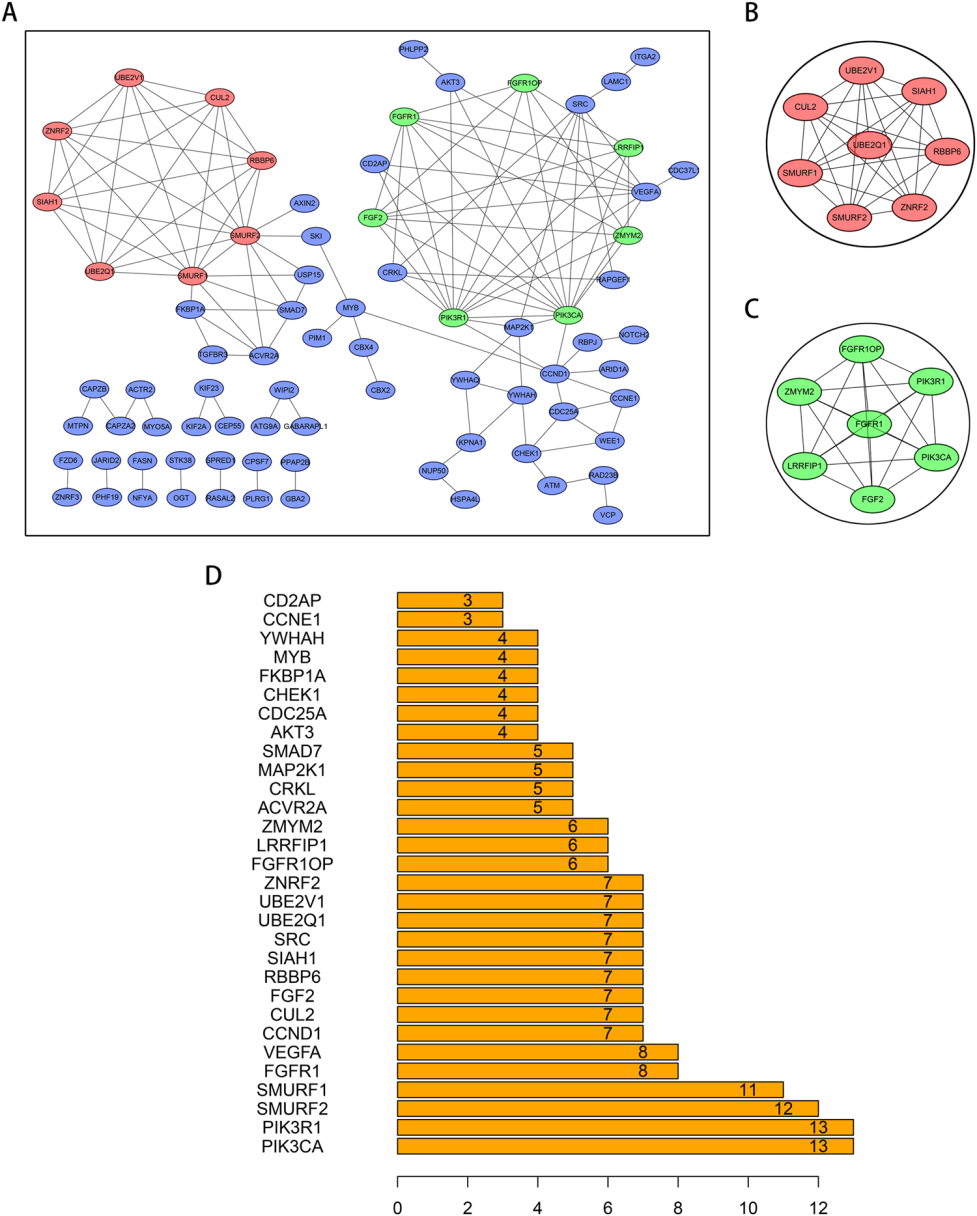
PPI network and modular analysis of the target genes. (**A**) Using the STRING online database, 78 target genes were filtered into the target genes PPI network complex. The red and green ovals were the target genes comprising the two most significant modules. (**B**) Module 1 consists of 8 nodes and 28 edges. (**C**) Module 2 consists of 7 nodes and 21 edges. (**D**) The target genes that have more than 2 edges associated with other node proteins.

The STRING (a database of known and predicted protein interactions)^17^ and Cytoscape were used to predict protein interactions among the target genes of five-miRNA signature. First, the target genes were submitted to the STRING web site to get protein–protein interaction (PPI) data. Then, the PPIs with combined scores greater than 0.900 were selected for constructing PPI networks, and disconnected nodes in the network were hidden. Total of 78 of the 219 target genes were filtered into the target genes PPI network complex, containing 78 nodes and 133 edges (Fig. 6A), and an additional 141 of the 219 target genes fall into the target genes PPI network. Among the 78 nodes, 15 central node genes were identified by filtering for a degree > 7 (each node had more than 7 connections/interactions). According the degree of importance, we chose 2 significant modules from the PPI network complex for further analysis using Cytoscape MCODE (Fig. 6B,C). Figure 6D shows that most significant 6 node degree genes were PIK3CA, PIK3R1, SMURF2, SMURF1, FGFR1 and VEGFA.

### Functional analysis of the prognostic five-miRNA signature via co-expressed target genes

**Figure 7:**
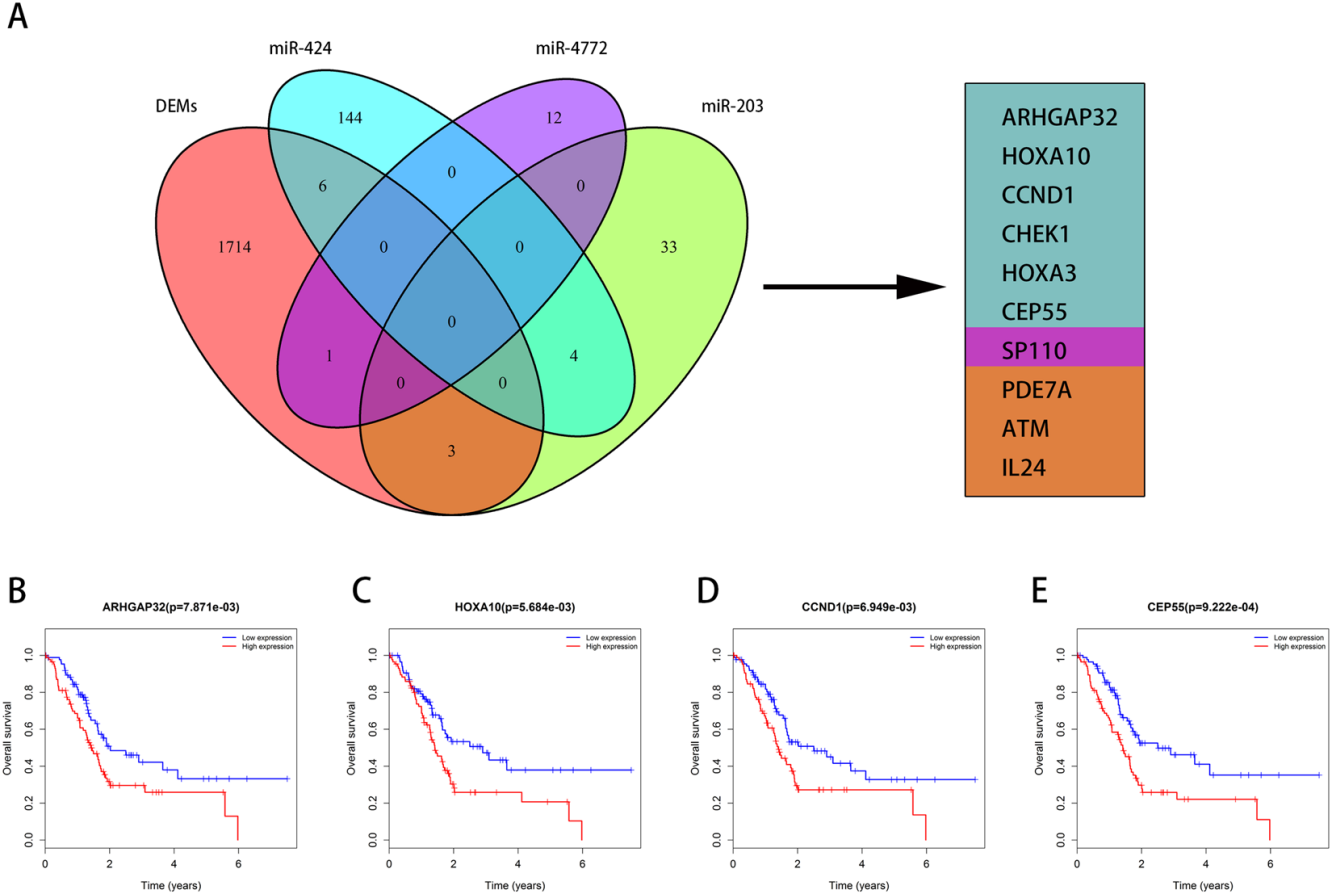
Co-expression analysis of the target genes. (**A**) Total of 10 overlapping target genes were co-expressed with the five-miRNA signature. Four overlapping target genes that associated with OS. (**A**) ARHPAG32. (**B**) HOXA10. (**C**) CCND1. (**D**) CEP55.

In order to explore the functional significance of the five-miRNA signature in pancreatic adenocarcinoma, we focused on the target genes that are co-expressed with the miRNA signature. In our previous study, we identified 1724 differentially expressed mRNAs (DEMs). From the list of target genes identified above, 10 were among the DEMs (Fig. 7A). After the OS analysis of these 10 target genes, we found that 4 were OS-associated genes in pancreatic adenocarcinoma: ARHPAG32 (Fig. 7B), HOXA10 (Fig. 7C), CCND1 (Fig. 7D) and CEP55 (Fig. 7E). This implies that the five-miRNA signature might contribute to pancreatic adenocarcinoma via regulating these target genes.

### qRT-PCR verification

**Figure 8:**
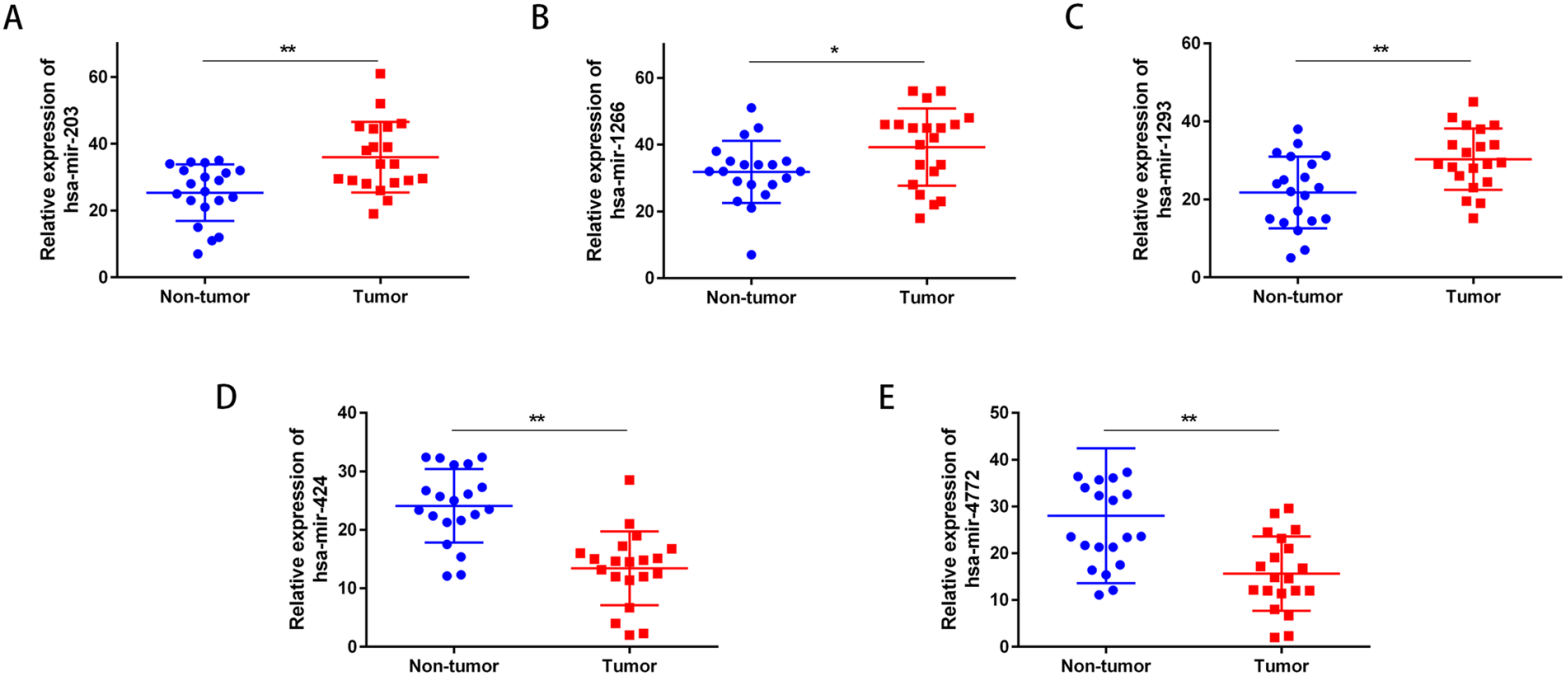
Aberrant expression of miRNAs in pancreatic adenocarcinoma. Expression of the five-miRNA signature was analyzed by qRT-PCR in pancreatic adenocarcinoma tissues. (**A**) miRNA-203. (**B**) miRNA-1266. (**C**) miR-1293. (**D**) miRNA-424. (**D**) miRNA-4772.

We examined the expression of the 5 specific miRNAs in the miRNA signature to test the reliability and validity of this signature in 20 newly diagnosed pancreatic adenocarcinoma patients using qRT-PCR. We applied the paired t-test to assess the differences between the pancreatic tumor tissues and the adjacent non-tumor pancreatic tissues. The results showed that miR-203, miR-1266 and miR-1293 were upregulated in pancreatic tumor tissues when compared with adjacent non-tumor pancreatic tissues, while miR-424 and miR-4772 were downregulated in pancreatic tumor tissues (Fig. 8). The results from this qRT-PCR validation in 20 newly diagnosed pancreatic adenocarcinoma patients were consistent with the above bioinformatics results. The qRT-PCR data revealed that our bioinformatics analysis was accurate.

## Discussion

In the present study, we have identified a five-miRNA signature consisting of hsa-miR-203, hsa-miR-424, hsa-miR-1266 hsa-miR-1293, and hsa-miR-4772. This signature was validated as being able to predict the clinical outcome of pancreatic adenocarcinoma based on RNA-seq technology. The differentially expressed miRNAs were identified by comparing data from pancreatic adenocarcinoma and normal tissues that was downloaded from TCGA database. The expression profiles of these miRNAs from 177 pancreatic adenocarcinoma patients were analyzed by univariate and stepwise multiple Cox proportional hazards regression analysis. The five miRNAs selected from this process were used to establish a predictive model based on the linear combination of these miRNAs. A distinct separation was observed in survival curves between patient groups with high-risk and low-risk scores using this predictive model. In addition, the ROC analysis achieved an AUC of 0.775, demonstrating sensitively and specificity of the five-miRNA signature model.

Ikenaga *et al*. reported that miRNA-203 was overexpressed in pancreatic adenocarcinoma samples and acted as a new prognostic marker for pancreatic adenocarcinoma^18^. Yabushita *et al*. found that miRNA-203 was significantly overexpressed in the serum of pancreatic adenocarcinoma rats compared to control rats^19^. Wang *et al*. showed that the expression levels of miR-424 were decreased in both esophageal squamous cell carcinoma tissues and cell lines^20^. However, Zhang *et al*. found that overexpression of miR-424 significantly increased cell migration and invasion, as well as the proliferation of colonies in non-small cell lung cancer^21^, which suggested it plays a complex role in cancer as it can act either as oncogene or tumor suppressor depending on the origin of cancer. Sevinc *et al*. suggested that high miR-1266 levels might be a significant prognostic factor for recurrence/metastasis and for the response to tamoxifen in estrogen receptor-positive breast cancer patients^22^. Luo *et al*. reported that miRNA-1293 acted as a prognostic biomarker of papillary renal cell carcinoma using the Cox ratio risk regression model^23^. Our results showed that miR-203, miR-1266 and miR-1293 were all upregulated in pancreatic adenocarcinoma and may therefore act as oncogenes in development of pancreatic adenocarcinoma. In addition, miR-203, miR-424 and miR-1293 can act as an independent prognostic factor in pancreatic adenocarcinoma, respectively. The function of miR-4772 in cancer was not clear. Future studies should focus on this point, and investigate the function of miR-4772 in pancreatic adenocarcinoma.

To gain an insight into the molecular functions of the five miRNAs, we predicted the target genes and analyzed the related pathways via GO annotation and KEGG enrichment analysis. The results showed that the prognostic miRNAs were involved in key biological processes such as cell proliferation, positive or negative regulation of transcription, the transforming growth factor beta receptor signaling pathway and enriched KEGG pathways including those in cancer, PI3K-Akt signaling pathway, and signaling pathways regulating pluripotent stem cells. To further explore the functions of the putative miRNA signature, we predicted PPI network of the target genes and found 4 OS-related target genes that are co-expressed with the five-miRNA. The TGF-β receptor signaling pathway causes both proliferative and tumor-suppressive responses depending on the biological context^24^. SMAD4 is one of the Smad family of signal transducers from TGF-β. It mediates pancreatic cell proliferation and apoptosis and is specifically inactivated in half of advanced pancreatic cancers^25^. The PI3K-Akt pathway is activated in many cancers, and inhibition of the PI3K-Akt pathway can induce cell apoptosis in most cancers. Mao *et al*. showed that inhibition of the PI3K-Akt pathway can induce cell apoptosis and reduce cell proliferation by downregulating Plk1 both *in vitro* and *in vivo*^26^. Abnormal signaling pathways play crucial roles in the pathogenesis and progression of pancreatic adenocarcinoma. Therefore, further investigations are needed to confirm these predictions, and to provide new therapeutic interventions in pancreatic adenocarcinoma.

Quantitative RT-PCR was used to examine the reliability and validity of expression of key miRNAs and the bioinformatics analysis. We test the 5 specific miRNAs in the miRNA signature (miR-203, miR424, miR-1266, miR-1293 and miR-4772), and validated the reliability of the results in 20 newly diagnosed pancreatic adenocarcinoma patients. The expression results were consistent with TCGA results.

However, there were a number of limitations in this study. First, the data used for the prognostic model in this study was from a single source (TCGA). The performance of this miRNAs signature might be more reliable if validation is performed with independent external data sets with long-term follow up. Second, the TCGA pancreatic adenocarcinoma cohort had a relatively high censored rate, which may affect the reliability of the Kaplan-Meier estimates. Therefore, further clinical studies validating the predictive efficacy of the signature and experimental research investigating the functions of the prognostic five-miRNA signature need to be conducted.

In conclusion, we identified a five-miRNA signature as a potential prognostic predictor for pancreatic adenocarcinoma patients. Further studies are needed to validate our findings with a large sample size, and further functional investigations are also required to explore the molecular mechanism of these miRNAs in pancreatic adenocarcinoma progression.

## Materials and Methods

### Patients and samples

Pancreatic adenocarcinoma tissues and matched adjacent normal pancreatic tissues were obtained from 20 patients who underwent surgery between Jan 2016 and Nov 2017 in Tongji Hospital, Wuhan, China. All samples were analyzed by two professional pathologists. The fresh tissue specimens were snap frozen in liquid nitrogen and then stored at −80 °C prior to RNA isolation. There was no pre-operative treatment prior to surgery. All patients signed the informed consent before surgery. This study was approved by the Human Ethics Committee of Tongji Hospital at Huazhong University of Science and Technology University (Wuhan, China) and carried out in accordance with the Declaration of Helsinki.

### RNA sequence data processing and screening of differentially expressed miRNAs

The raw sequencing data and clinical information were downloaded from TCGA database (https://cancergenome.nih.gov/). The detail of data processing and screening of differentially expressed miRNAs or mRNAs were described in our previous study.

### Survival analysis and definition of miRNA-related prognostic signature

The association between the expression of differentially expressed miRNAs and patient overall survival was evaluated by univariate Cox proportional hazards regression analysis using the survival R package. Only those miRNAs with a p-value < 0.05 were considered as candidate variables and entered into a stepwise multivariate Cox regression analysis.

### Risk stratification and ROC curve

According to the predictive miRNA signature model, the risk score for each of the 177 patients was calculated. The patients were then classified into high-risk or low-risk group using the median risk score as the cutoff value. Overall survival curves were generated using the Kaplan-Meier method, and two-sided log-rank tests were employed to compare the differences in overall survival time between the high-risk and low-risk patient groups. The sensitivity and specificity of the miRNA prognostic signature to predict clinical outcome were evaluated by calculating the area under curve (AUC) of the receiver operating characteristic (ROC) curve using the R package^27^.

### Target prediction of miRNA and functional enrichment analysis of GO annotation and KEGG signaling pathway

The target genes of the five-miRNA (miR-203, miR-424, miR-1266, miR-1293, and miR-4772) were predicted using TargetScan, miRDB, miRTarBase, and miRanda online analysis tools. To further enhance the reliability of the bioinformatic analysis, the overlapping target genes were identified using a Venn diagram. Using the DAVID and KOBAS online databases, the functional analysis of the GO annotation and KEGG signaling pathways were carried out. In the GO analysis, the categories included biological process (BP), cellular component (CC) and molecular function (MF) terms, and a p-value < 0.05 was regarded as statistically significantly different. To add quantitative molecular information to the GO terms of interest, we used ggplot2 package^28^, which permitted the incorporation of data derived from expression level measurements with those obtained from the functional annotation enrichment analysis. In the KEGG pathways analysis, enriched pathways were identified according to the hypergeometric distribution with a p-value < 0.05, and were performed with clusterProfiler package^29^. In addition, to provide a readable graphic representation of the complex relationship between target genes and relative KEGG pathway, the “pathway-gene network” was constructed using Cytoscape.

### Protein–protein interaction network construction by STRING

Using the ID number of the miRNA target genes, the products of the 219 target genes in the network were analyzed by the STRING online tool to predict the interactions among them. A combined score of <0.900 (highest confidence score) was considered significant. The hub protein was selected based on its association with other proteins. The target genes with numerous associations with other target genes suggested important roles in the protein–protein interaction network.

### Total RNA extraction and quantitative real-time PCR (qRT-PCR) verification of miRNA expression

Total RNA was isolated with TRIzol reagent(Invitrogen, Carlsbad, CA, USA) according to the manufacturer’s protocol, and RNA purity was measured using a NanoDrop 2000 spectrometer (Thermo Fisher Scientific, Waltham, MA, USA). Reverse transcription reactions using the A3500 reverse transcription system kit (Promega, Madison, WI, USA) were conducted in two steps according to the manufacturer’s protocol.qRT-PCR analysis the expression of the five miRNAs was performed on a LightCycler 480 (Roche Life Science, Indianapolis, IN, USA) and Agilent 2100 Bioanalyzer (Agilent Technologies, Santa Clara, CA, USA) using a TaqMan miRNA Assay according to manufacturer’s protocol (Applied Biosystems, Foster City, CA, USA). All reactions were run in triplicate. Expression of the five miRNAs was normalized to the expression level of the housekeeping gene U6.

### Statistical analysis

Statistical analysis was performed using R version 3.4.1. The data were expressed as the mean ± standard deviation (SD), and statistically compared by performing the paired t-test. A p-value < 0.05 was considered statistically significant.

## Electronic supplementary material

Supplemental Table1

Supplemental Table2
